# Infarto Agudo do Miocárdico: Precisamos de Marcadores para Lesão de Microcirculação? E a Relação Fibrinogênio/Albumina Seria a Resposta?

**DOI:** 10.36660/abc.20230809

**Published:** 2024-02-19

**Authors:** Ricardo Wang, Fernando Carvalho Neuenschwander

**Affiliations:** 1 Instituto Orizonti Belo Horizonte MG Brasil Instituto Orizonti, Belo Horizonte, MG – Brasil; 2 CardioOne Clinic Belo Horizonte MG Brasil CardioOne Clinic, Belo Horizonte, MG – Brasil; 3 Hospital das Clínicas da Universidade Federal de Minas Gerais Belo Horizonte MG Brasil Hospital das Clínicas da Universidade Federal de Minas Gerais (UFMG), Belo Horizonte, MG – Brasil

**Keywords:** Infarto do Miocárdio, Mortalidade, Reperfusão Miocárdica, Stents, Fibrinolíticos, Albumina, Fenômeno de Não Refluxo

O infarto agudo do miocárdio (IAM) é a principal causa de morte no mundo, e a segunda causa de morte no Brasil. O maior avanço no seu tratamento foi a terapia de reperfusão, seja ela medicamentosa, através do uso de fibrinolíticos ou por angioplastia coronariana com implante de stents. Este último, além da superioridade na qualidade da reperfusão, tem se demostrado ser tempo sensível, quão mais precoce a intervenção maior é a redução nos desfechos cardiovasculares, tanto no contexto da Síndrome Coronariana Aguda sem Supra do Segmento ST e principalmente com elevação do segmento ST.^1^

No contexto do IAM com supra do segmento ST (IAMCSST), o tempo entre os sintomas e a reperfusão pela angioplastia primária, é considerado o principal redutor de morte cardiovascular, levando a preservação da função ventricular e recuperação mais precoce.

Mesmo sendo tratada precocemente, uma parcela desses pacientes irá experimentar uma reperfusão incompleta, caracterizada por disfunção da microcirculação acarretando a redução blush miocárdico, da velocidade em que o contraste percorre o leito coronário, podendo chegar ao extremo que é o fenômeno de "no Reflow" (falta de fluxo, mesmo com a artéria aberta).

Nesta edição dos Arquivos Brasileiros de Cardiologia, Kaplangoray et al.,^2^ demonstraram que a relação Fibrinogênio/Albumina (F/A) pode ser um bom preditor de uma reperfusão incompleta, sendo assim, poderia ser utilizado como marcador para este evento. Neste estudo, ressaltamos que a população que apresentava maior relação F/A eram: pacientes mais idosos, com maior prevalência de diabéticos e lesões coronarianas mais complexas (Syntax Score). Após realizar ajustes, por meio da análise multivariada, a relação F/A permaneceu como fator de risco isolado para redução da perfusão microvascular.

Apesar dos autores elegantemente terem discutido o racional teórico para utilização relação F/A, demonstrando todo embasamento teórico para sua utilização, consideramos desafiadora sua incorporação na prática clínica. Para utilização rotineira de um marcador algumas perguntas devem ser respondidas: 1. O marcador auxilia no diagnóstico da condição clínica? 2. O marcador é preditor de desfechos clínicos de interesse? 3. É fácil de ser utilizado na prática clínica? 4. É possível intervir de forma diferente a partir dos resultados encontrados e consequentemente reduzir eventos? 5. Qual é o impacto financeiro para sua implementação?

Pensando na relação fibrinogênio/albumina, temos dúvidas de sua utilidade clínica: Não auxilia no diagnóstico, pois para o paciente com quadro de IAMCSST, a prioridade seguirá sendo o diagnóstico precoce, baseado na clínica e nas alterações eletrocardiográficas, seguida de terapia de reperfusão no menor tempo possível. Neste contexto, durante os vários processos que correm simultaneamente, é feita a coleta de amostra de sangue, entretanto estes exames raramente estão prontos antes da realização da angioplastia coronária e esperar acarretará o atraso do procedimento, e sem os resultados nada de diferente poderá ser executado durante a intervenção. Portanto, a não reperfusão da microcirculação vai continuar por meio da angiografia e o eletrocardiograma pós procedimento. Outro ponto importante a considerar é o tamanho da amostra. Os desfechos substitutivos poderiam se traduzir em aumentos dos desfechos cardiovasculares duros (mortalidade principalmente)?^3^

Algumas hipóteses permanecem abertas: e se tivermos disponível um *kit fast* para realização desse teste? Haveria mudança na abordagem da angioplastia primária? Uso de fibrinolíticos, inibidores de glicoproteína IIb/IIIa pré procedimento (para reduzir o fibrinogênio)? Infusão de albumina? Infelizmente, até o presente momento não possuímos evidências robustas para responder estas questões. Neste contexto, podemos dizer que a medida da relação F/A encontra-se no campo de geração de hipóteses, e necessita de mais estudos para responder a todas essas perguntas, antes de ser incorporada na prática clínica. Sem isso, esse marcador corre o risco de cair no esquecimento.
