# Supplementary material for: Infarto Agudo do Miocárdico: Precisamos de Marcadores para Lesão de Microcirculação? E a Relação Fibrinogênio/Albumina Seria a Resposta?
Source: Arq Bras Cardiol. 2024 Feb 19;120(11):e20230809. [Article in Portuguese] doi: 10.36660/abc.20230809 (PMC11098586; doi:10.36660/abc.20230809)
Supplement: Translation

# Translation: Acute Myocardial Infarction: Do We Need Markers for Microcirculation Injury? Moreover, Would the Fibrinogen/Albumin Ratio Be the Answer?

**Authors:** Ricardo Wang, Fernando Carvalho Neuenschwander

**Affiliations:** 1 Belo Horizonte MG Brazil Instituto Orizonti, Belo Horizonte, MG – Brazil; 2 Belo Horizonte MG Brazil CardioOne Clinic, Belo Horizonte, MG – Brazil; 3 Belo Horizonte MG Brazil Hospital das Clínicas da Universidade Federal de Minas Gerais (UFMG), Belo Horizonte, MG – Brazil

**Keywords:** Myocardial Infarction, Mortality, Myocardial Reperfusion, Stents, Fibrinolytic Agents, Albumins, No-Reflow Phenomenon

Acute myocardial infarction (AMI) is the leading cause of death in the world and the second cause of death in Brazil. The biggest advance in its treatment was reperfusion therapy, be it medication, through the use of fibrinolytics or coronary angioplasty with stent implantation. The latter, in addition to the superiority in the quality of reperfusion, has been shown to be time-sensitive; the earlier the intervention, the greater the reduction in cardiovascular outcomes, both in the context of Acute Coronary Syndrome without ST-Segment Elevation and especially with ST^1^ segment elevation.

In the context of ST-segment elevation AMI (STEMI), the time between symptoms and reperfusion by primary angioplasty is considered the main reduction in cardiovascular death, leading to the preservation of ventricular function and earlier recovery.

Even if treated early, a portion of these patients will experience incomplete reperfusion, characterized by microcirculation dysfunction, resulting in a reduction in myocardial blush, the speed at which the contrast travels through the coronary bed, and can reach the extreme that is the "no-Reflow" phenomenon (lack of flow, even with the artery open).

In this edition of *Arquivos Brasileiros de Cardiologia*, Kaplangoray et al.^2^ demonstrated that the Fibrinogen/Albumin (F/A) ratio can be a good predictor of incomplete reperfusion; therefore, it could be used as a marker for this event. In this study, we emphasize that the population with the highest F/A ratio were older patients with a higher prevalence of diabetics and more complex coronary lesions (Syntax Score). After making adjustments through multivariate analysis, the F/A ratio remained an isolated risk factor for reduced microvascular perfusion.

Although the authors elegantly discussed the theoretical rationale for using the F/A ratio, demonstrating all theoretical basis for its use, we consider its incorporation into clinical practice challenging. For routine use of a marker, some questions must be answered: 1. Does the marker help in diagnosing the clinical condition? 2. Is the marker a predictor of clinical outcomes of interest? 3. Is it easy to use in clinical practice? 4. Is it possible to intervene differently based on the results found and consequently reduce events? 5. What is the financial impact of its implementation?

Thinking about the fibrinogen/albumin relationship, we have doubts about its clinical usefulness: It does not help in the diagnosis because, for the patient with STEMI, the priority will continue to be early diagnosis, based on clinical and electrocardiographic changes, followed by reperfusion therapy in the shortest possible time. In this context, during the various processes that run simultaneously, a blood sample is collected; however, these tests are rarely ready before the coronary angioplasty is performed, and waiting will result in a delay in the procedure, and without the results, nothing different can be carried out during the intervention. Therefore, non-reperfusion of the microcirculation will continue through angiography and post-procedure electrocardiogram. Another important point to consider is the sample size. Could surrogate outcomes translate into increases in hard cardiovascular outcomes (mainly mortality)?^3^

Some hypotheses remain open: what if we have a fast kit available to carry out this test? Would there be a change in the approach to primary angioplasty? Use of fibrinolytic, glycoprotein IIb/IIIa inhibitors pre-procedure (to reduce fibrinogen)? Albumin infusion? Unfortunately, to date, we do not have robust evidence to answer these questions. In this context, we can say that the measurement of the F/A ratio is in the field of generating hypotheses and requires further studies to answer all these questions before being incorporated into clinical practice. Without this, this marker risks falling into oblivion.
